# Primary Pleomorphic Undifferentiated Sarcoma—a Rare Renal Localization: A Case Report

**DOI:** 10.1155/2012/862493

**Published:** 2012-11-19

**Authors:** Soufiane Mellas, Ahmed Amine Bouchikhi, Mohamed-Fadl Tazi, Abdelhak Khallouk, Jallal-Eddin Elammari, Mohamed-Jamal El Fassi, Naoufal Mellas, Moulay Hassan Farih

**Affiliations:** ^1^Department of Urology, Hassan II University Hospital of Fez, Fez 30020, Morocco; ^2^Department of Anatomy, Faculty of Medicine of Fez, Fez, Morocco; ^3^Department of Oncology, University Hospital of Fez, Fez, Morocco

## Abstract

Undifferentiated pleomorphic sarcoma is known as a soft tissue sarcoma. Very few cases of this tumor originating from the renal parenchyma or renal capsule have been reported. We report a case of a 70-year-old patient admitted for enormous ureterohydronephrosis and pyelonephritis due to a pelvic ureter lithiasis. After draining by ureteral double J catheter, a nephroureterectomy was performed for nonfunctional kidney confirmed by scintigraphy. The histopathological study shows a pleomorphic undifferentiated sarcoma. The patient was sent to oncologists. Chemotherapy was proposed but the family decided to stop the treatment. The patient passed away 10 months later. Clinicians and pathologists should be aware of the very low occurrence of this renal tumor, which is extremely rare. Currently there is no consensus about its management. Our case extends the literature concerning this tumor.

## 1. Introduction

Undifferentiated pleomorphic sarcoma represents up to 10% of all adult soft tissue sarcomas (STSs) [1], this tumor is defined as a mesenchymal neoplasma composed of spindle and pleomorphic cells in varying proportions [1]. Pleomorphic sarcoma originating from renal parenchyma or capsule is extremely rare and constitutes less than 6% of renal sarcomas [2]. Here we report a case of pleomorphic sarcoma discovered after nephroureterectomy in nonfunctional kidney patient.

## 2. Case Report

**Figure 1:**
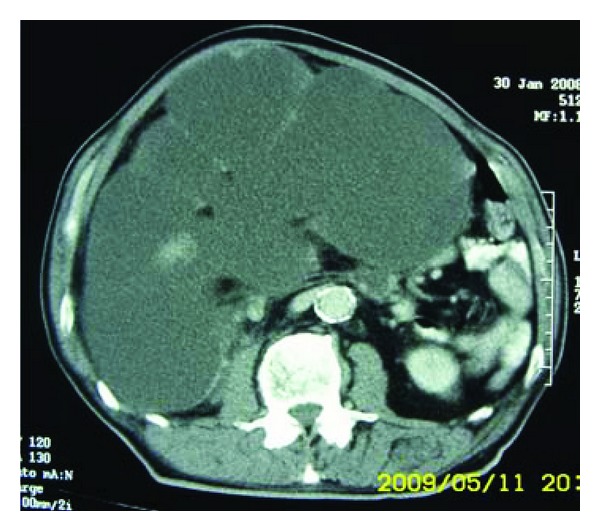
CT of the abdomen showing giant hydronephrosis.

**Figure 2:**
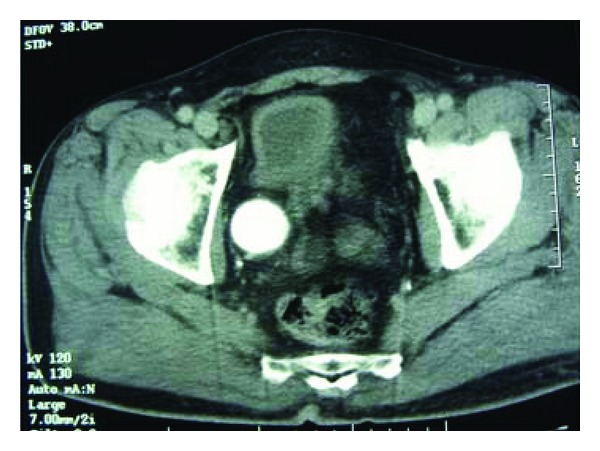
CT of the same patient showing pelvic ureteral stone.

**Figure 3:**
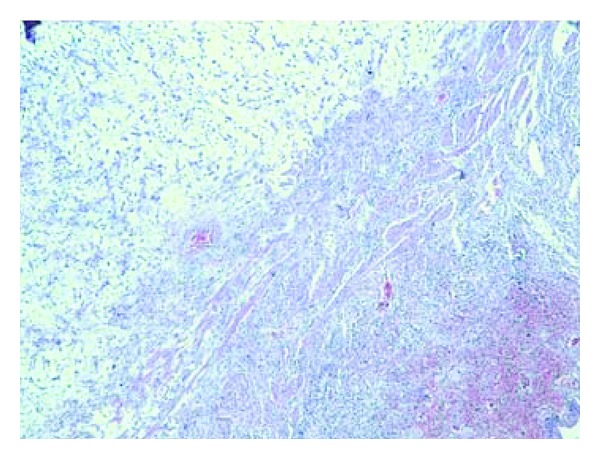
HESX4: sarcomatous tumoral proliferation arising from renal tissue.

**Figure 4:**
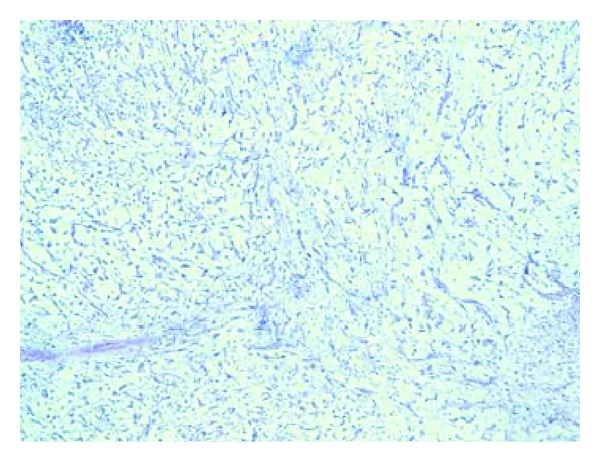
HESX10: atypical spindle cells proliferation arranged in a myxoid background.

A 70-year-old Moroccan patient was admitted in emergency unit for flank pain and fever. He complained of pain in the right upper abdomen in the last 2 years associated to loss of weight and voiding difficulty. However, there was no history of hematuria. The clinical examination revealed an enormous nonmobile mass in the right lumbar region. Routine investigations showed anemia (hemoglobin 5 g/dL). Biochemical studies were within normal limits. Ultrasonography performed on the patient revealed a big transsonic masse hiding the right kidney. Computer-assisted tomography of the abdomen demonstrated a mass of an enormous hydronephrosis due to a big stone measuring 37 mm diameter in the pelvic ureter (Figures 1 and 2). The patient initially underwent drainage by a double J-ureteral stent and transfusion. A radioisotope nephrography was performed two weeks later and showed a right kidney functional rate of less than 5%. One month later, nephroureterectomy was performed with a stone removal by transperitoneal laparotomy. The histopathological study confirmed a sarcomatous tumor proliferation made by atypical spindle cells arranged in a myxoid background arising from renal parenchyma (Figures 3 and 4). The patient was referred to oncology department that suggested chemotherapy. Once the patient was informed by the disease prognosis, the family decided to stop treatment. The patient died ten months later.

## 3. Discussion

Pleomorphic sarcoma or undifferentiated high-grade spindle sarcoma is now the preferred term to designate high-grade soft tissue sarcomas failing to show any specific line of differentiation using currently available ancillary techniques [1]. This pathology occurs most frequently in the limbs of middle to advanced-age adults with a male predominance [1]. In general, primary renal sarcomas are rare tumors and represent up to 3% of all renal malignancies [2]. Pleomorphic sarcoma arising from renal parenchyma or capsules is extremely rare and constitutes less than 6% of all renal sarcomas [2]. Only 29 cases have been reported in the literature [3]. There are no specific clinical features of pleomorphic sarcoma, while abdominal mass and abdominal pain are the most revealed symptoms and hematuria is rarely associated [3–5]. A preoperative radiologic diagnosis based on CT-scan of pleomorphic sarcoma is not possible. Hence, simple or radical nephrectomy with a suspicion of renal cell carcinoma is usually undertaken. Thus, histopathological examination remains the unique diagnostic modality allowing final diagnosis of this rare entity [2].

There are no specific criteria allowing the diagnosis of primary renal pleomorphic sarcoma. However, it has been suggested that [3]

1. there should not be any history of sarcoma elsewhere to exclude metastasis;
2. the gross appearance must be compatible with origin in the kidney;
3. sarcomatoid renal cell carcinoma must be excluded;
4. metastasis, if present, should be smaller than the renal tumor.

Pleomorphic sarcoma has complex karyotypes. No specific structural or numerical abnormalities have been proved to be useful for identification purposes [2].

The histological differential diagnoses of this tumor include other malignant and benign renal lesions benign. The unique curative treatment is the radical surgical excision [6]. An incomplete resection would yield a poor prognosis leading to local recurrence and/or metastasis particularly in the lungs and lymph nodes [7]. This aggressive tumor demonstrated that 30 to 50% of patients might die within 5 years after diagnosis [1, 3, 6–9].

The small number of cases assessed so far does not allow ultimate evaluation of the role of adjuvant chemotherapy and/or and radiotherapy [9]. However, this tumor is chemosensitive, particularly to doxorubicin- and ifosamide-based treatment [9].

## 4. Conclusion

Primary renal pleomorphic undifferentiated sarcoma is an extremely rare tumor without any distinctive clinical or imaging features. Wide excision followed by radiotherapy is the recommended treatment. Adjuvant chemotherapy may be considered according to the profile of the patient.
